# Evaluation of a Translatable Web-Based Intervention for Increasing Physical Activity Among Cancer Survivors: Pilot Randomized Trial

**DOI:** 10.2196/79610

**Published:** 2025-10-02

**Authors:** Jessica L Unick, Christine Duffy, Don Dizon, Mary Ann Fenton, Zihuan Cao, Katrina Oselinsky, Selene Y Tobin, Rena R Wing

**Affiliations:** 1 Weight Control and Diabetes Research Center, Miriam Hospital, 196 Richmond St, Providence, RI, 02903, United States, 1 4017938966; 2 Warren Alpert Medical School, Brown University, Providence, RI, United States; 3 Brown Physicians Group, Providence, RI, United States; 4 Lifespan Cancer Institute, Providence, RI, United States; 5 Rhode Island Hospital, Providence, RI, United States; 6 Anshutz Medical Campus, University of Colorado, Aurora, CO, United States

**Keywords:** eHealth, exercise, physical activity, survivorship, behavioral intervention

## Abstract

**Background:**

Cancer survivors face long-term health challenges posttreatment. Physical activity (PA) can help manage cancer-related side effects and offer additional health benefits, yet up to 80% of survivors do not meet PA guidelines. Effective and translatable PA interventions are needed.

**Objective:**

This randomized trial assessed the feasibility, acceptability, and preliminary efficacy of a 12-week automated Internet program for increasing moderate-to-vigorous physical activity (MVPA) among cancer survivors. A secondary aim examined the effect of the intervention on physical and mental well-being.

**Methods:**

Inactive (<60 min/wk of PA) cancer survivors who completed cancer-directed treatment in the past 3‐12 months or those on a stable maintenance treatment regimen were randomized to the Energize! Exercise Program or Newsletter control condition. The Energize! Program was fully automated and involved weekly behaviorally-based video lessons, homework assignments, exercise planning and reporting, and progressive MVPA goals (75 to 200 min/wk). Algorithm-generated personalized feedback was provided based on PA goal attainment and homework completion. The newsletter group received bimonthly PA education newsletters (a total of 6). Assessments occurred at baseline, 3 months (postintervention), and 6 months (following a 3-month no-contact follow-up). Feasibility was assessed via enrollment and retention rates, acceptability was assessed via intervention engagement metrics and program satisfaction questionnaire, and MVPA was assessed via both self-report and accelerometer (min/wk of total and “bouted” MVPA [accumulated in bouts ≥10 min]). Health-related outcomes (eg, quality of life, fatigue, psychological distress, psychological symptoms, and fear of cancer recurrence) were assessed via electronic questionnaires.

**Results:**

Forty-six adults aged 55.2 (SD 8.3) years, with BMI mean 33.0 (SD 7.6) kg/m²; 42 (91.3%) female, and 37 (80.4%) non-Hispanic White enrolled in this trial. Feasibility metrics indicate that 69% (46/67) of those who screened eligible were randomized and 6-month retention among randomized participants was 94% (43/46). Acceptability was also high, as evidenced by the percentage of lessons viewed (mean 87.7%, SD 21.3%), exercise plans submitted (mean 82.6%, SD 25.8%), homework assignments completed (mean 77.2%, SD 25.2%), and weeks in which exercise minutes were logged (mean 85.9%, SD 22.1%). Program satisfaction ratings were higher in Energize (mean 5.8, SD 1.6; 1‐7 scale) versus Newsletter (mean 3.2, SD 1.6; *P*<.001). Energize! increased self-reported (92.7 min/wk), bouted (35.4 min/wk), and total (46.3 min/wk) MVPA at 3 months (Cohen *d*=0.74‐0.94), and these changes were partially maintained at 6 months. Increases in MVPA were smaller among Newsletter participants (*d*=0.28‐0.47). Group differences in health-related outcomes were minimal and mixed, favoring Energize! over Newsletter for vitality (*d*=0.63) and somatization (*d*=0.76) at 3 months, and for depression (*d*=0.59) and anxiety (*d*=0.51) at 6 months.

**Conclusions:**

The automated Energize! Program is feasible, acceptable, and associated with positive changes in MVPA, yet future studies are needed to improve MVPA long-term. Findings suggest that self-guided PA programs may be beneficial for increasing MVPA among cancer survivors.

## Introduction

Early detection and improved treatments have significantly reduced cancer mortality rates in recent years, resulting in greater numbers of cancer survivors [1]. Currently, there are over 18.1 million cancer survivors in the United States, and it is estimated that rates of cancer survivorship will increase by an additional 44% by 2040 [2]. While survivorship rates are encouraging, cancer survivors continue to face unique health challenges (eg, limitations in daily activities, anxiety, depression, fear of recurrence, and persistent fatigue [3-7]) which can persist well beyond the end of cancer treatment and reduce physical and mental health-related quality of life.

Physical activity (PA) is a modifiable lifestyle behavior that can help ameliorate these adverse health effects and is positively associated with numerous physiological and psychological health benefits. Findings from the American College of Sports Medicine’s International Multidisciplinary Roundtable indicate that there is strong evidence that regular PA can improve common cancer-related side effects, which include anxiety, depression, fatigue, and reductions in health-related quality of life, physical functioning, and cardiorespiratory fitness [8,9]. PA is also associated with a lower risk of all-cause mortality, cancer recurrence, and the likelihood of other comorbid chronic diseases (eg, type 2 diabetes and cardiovascular disease) [10-12]. However, estimates suggest that only 17% to 58% of cancer survivors achieve the national PA guideline of ≥150 minutes/week of moderate-intensity PA [13-19]. Thus, translatable interventions for increasing PA among the growing number of cancer survivors are needed.

Automated, Internet-delivered PA programs, which are a type of eHealth intervention, allow for wide-scale dissemination at a relatively low cost. Pew Research data indicate that 95% of Americans use the Internet [20], making it an ideal medium for delivering behaviorally-based PA programs, as it can reduce provider costs and reach large numbers of individuals. Further, the cost of delivering an automated Internet program is low, and there is no added cost for enrolling additional participants. This is particularly important from a translation perspective, as this type of program does not need to be delivered by exercise physiologists, physical therapists, rehabilitation professionals, or physicians. Moreover, patients are not required to live near a treatment or rehabilitation center and can access the entire intervention on their own, without time constraints, travel barriers, or schedule limitations (eg, sessions only offered during certain hours at specific facilities).

Although eHealth interventions are widely used to promote PA and other lifestyle behaviors in “non-clinical” settings or within certain chronic disease populations (eg, cardiovascular disease and diabetes), application of these technologies to promote PA among cancer survivors has lagged behind [21]. Considerable progress has been made in the past several years; however, there remains a shortage of randomized controlled trials, with stringent investigative processes, evaluating the effects of behavioral eHealth interventions among cancer survivors [22-24]; additional research is clearly warranted.

This randomized trial examines the feasibility and acceptability of a 12-week, fully automated Internet program for increasing PA among cancer survivors and explores the effect of this program on PA relative to a newsletter control condition. Specifically, it uses rigorous methodology (eg, a randomized trial and PA assessment via accelerometers), incorporates essential behavior change techniques (eg, goal setting, action planning, self-monitoring, problem-solving, and affect regulation strategies), and examines whether intervention effects are sustained 3 months postintervention, thereby addressing important deficits in the current body of research. A secondary aim of this trial was to compare treatment groups on measures of physical and mental well-being over the entire study period.

## Methods

### Participants and Recruitment

To be eligible, individuals needed to have a confirmed cancer diagnosis (excluding nonbasal or squamous cell carcinoma) and have either completed all cancer-directed treatment in the past 3‐12 months or be on a maintenance treatment regimen for which they have been stable for at least 3 months. Other eligibility criteria included being 18‐70 years of age, a BMI of 18.5 to <45 kg/m^2^, daily Internet access, English speaking, and inactive, defined as engaging in <60 minutes/week of self-reported moderate-intensity PA over the past 3 months. Exclusion criteria included current, recent, or planned pregnancy over the next 6 months, recent (< 2 y) hospitalization for a psychiatric condition, or the presence of a medical condition for which PA is contraindicated. These broad inclusion criteria were selected to enhance the generalizability of study findings and not limit enrollment to a particular cancer type.

Participants were recruited via advertisements at local oncology clinics in the greater Providence, Rhode Island area, and via national social media advertisements (eg, Facebook). Interested individuals could scan a QR code on physical flyers or click on a link within a social media advertisement to be directed to an electronic screener. This screener was designed to assess initial eligibility by confirming that age, BMI, ability to exercise, and PA criteria were met. Individuals deemed initially eligible based upon the electronic screener were then contacted by a member of the research team, and a more thorough eligibility screener was conducted via telephone. Those who continued to be eligible were invited to a one-on-one orientation session to learn more about the study, ask questions, and obtain informed consent. These sessions were conducted in-person or via videoconference (eg, Zoom).

### Study Procedures and Randomization

Cancer survivors enrolled in this study and randomized to receive the 12-week Energize! Exercise Program or a Newsletter control condition. Randomization occurred at baseline using sealed, opaque envelopes containing pregenerated allocation assignments, stratified by maintenance therapy status (on vs off therapy) to ensure balance across conditions. Envelopes were prepared in advance, sequentially numbered, and opened in order of enrollment. Assessments, which consisted of electronic questionnaires and PA measurement, occurred at baseline, 3 months (immediately postintervention), and 6 months (following a 3-month, no-contact follow-up period). Descriptions of each assessment measure and treatment condition are provided in greater detail below.

### Primary and Secondary Aims

The primary aims of this study were to assess the feasibility, acceptability, and preliminary efficacy of the Energize! Exercise Program for improving MVPA among cancer survivors, relative to a Newsletter control condition. Feasibility was evaluated via retention and enrollment rates. Of particular interest were the conversion rates of (1) those who completed the screening process who were deemed eligible, and (2) those who screened eligible but were eventually randomized. Acceptability was assessed via engagement with the intervention (eg, percentage of video lessons viewed, exercise plans submitted, homework assignments completed, and weeks in which exercise minutes were logged), and via the program satisfaction questionnaire. No predetermined thresholds for feasibility or acceptability were established before the study began. Changes in MVPA were assessed via both self-report and accelerometer (described in detail below). Secondary aims were to examine the effect of the intervention on health-related physical and mental well-being, which were assessed via a series of electronic questionnaires. This trial sought to enroll approximately 50 participants; however, no formal power calculations were conducted given the preliminary nature of this study.

### Energize! Exercise Program

Participants randomized to the intervention group received the 12-week Energize! Exercise Program. This fully automated, behaviorally-based, Internet program was designed to increase moderate-intensity PA to a level consistent with national recommendations. The entire program was delivered via a study website, which could be accessed via any web browser on a computer, tablet, or smartphone. No human intervention contact was provided. Each week, participants were given a prescribed exercise goal and asked to submit an exercise plan, watch a multimedia lesson, complete a brief homework assignment, and report their exercise on the study website. Computer-generated, personalized feedback messages were also provided weekly. Each of the Energize! Program components is described in greater detail below.

#### Exercise Goals

Participants were given a weekly, moderate-intensity PA goal that started at 75 minutes/week and increased by 25 minutes/week every other week, until reaching 200 minutes by week 11. Given that this program was designed to increase “purposeful” moderate-intensity aerobic exercise (eg, brisk walking and cycling), participants were instructed not to count activities such as hatha yoga, strength training, household chores (eg, gardening or vacuuming), or occupational activities (eg, restaurant server and postal worker), toward their weekly aerobic PA minute goal. To promote a regular habit of PA, participants were encouraged to exercise 5 days/week.

#### Exercise Planning

Beginning at week 2, participants were encouraged to plan their exercise prior to the start of each week and submit that detailed exercise plan via the study website. Specifically, individuals were asked to consider their schedule for the upcoming week and record when they planned to exercise (ie, day of week and time of day), as well as the type and duration of exercise they planned to do.

#### Multimedia Lessons

Each week, participants were instructed to watch a 10‐ to 15-minute video lesson. These lessons were designed to teach behavioral principles for modifying PA behavior. During week 1, a general overview of the program was provided along with an exercise prescription. Other video lesson topics included exercise planning, managing negative thoughts, stimulus control, overcoming exercise barriers, affect and exercise enjoyment, a midpoint check-in, managing exercise slips, exercise motivation, turning sitting time into active time, future-oriented mindsets, and thinking like an exerciser.

#### Homework Assignments

Participants were asked to complete weekly homework assignments electronically to help apply the content from the video lessons to their personal lives. Each assignment was designed to correspond to the lesson of the week and take no longer than 10 minutes to complete. Example assignments included the creation of an exercise routine to promote habit formation, journaling about the value of exercise and positive feelings associated with exercise, problem-solving around exercise barriers, and personal self-reflection based upon one’s progress in the program. No specific feedback was provided to the participant based on their responses to the homework assignments. However, as noted below, automated feedback, based solely upon whether the participant did or did not complete the homework assignment, was included as part of the weekly feedback message.

#### Self-Monitoring of Exercise

Similar to the exercise planning component of this program, participants were also asked to report all exercise performed on the study website daily. This included the time of day that the exercise was performed, the type of exercise, and the number of exercise minutes.

#### Automated Feedback Messages

Automated, yet personalized feedback was provided weekly based on the data input by the participant from the previous week. Twelve weeks of human-developed, tailored messages were embedded into the study website and took into consideration the following factors: (1) number of exercise minutes performed, (2) whether the homework assignment was completed or not, and (3) whether any injury, illness, or vacation prevented the participant from achieving their weekly exercise goal. Table 1 provides the response options and example feedback message for each factor. Generally, feedback messages were designed to be encouraging and motivational, praising individuals for meeting goals and providing support, encouragement, and specific behavioral recommendations for when goals were not met.

**Table 1.** Response options used to generate weekly, algorithm-derived feedback messages and example messaging for a given week.

Response category and response options	Example feedback message
Amount of exercise reported	
Response option a: Did not report any exercise	It looks like you didn’t report any exercise this week. We hope everything is OK. Perhaps something unexpected happened or you had a particularly busy week. Whatever the case, we hope you can get back on track! Consider the reasons you joined this program. Try to problem-solve around your current exercise barriers. Remember that when it comes to exercise, some exercise is always better than no exercise. You got this!
Response option b: Achieved exercise minute goal	You met the exercise goal for the week - great job! Hopefully, you’re starting to get into the swing of things and that exercise is automatically becoming part of your daily routine. The exercise goal will be increasing to 125 minutes this week. Start thinking about how you will get those additional 25 minutes of exercise in so that you can meet your goal again next week.
Response option c: Some exercise but less than half of goal	Meeting the exercise goal is an important part of this program. We are glad to see that you were able to get at least a little bit of exercise this week. Challenge yourself next week to make exercise a priority. To help you achieve your exercise goal, you could consider adding a positive social cue. Ask a friend, coworker or family member to join you on a walk, hike or bike ride. This is a great strategy for adding additional accountability and for making exercise fun and social. Keep pushing yourself this week to reach the goal. We know that you can do it!
Response option d: Did not achieve goal, but exercised more than half of the total goal minutes	Meeting the exercise goal is an important part of this program. We are thrilled to see that you’re putting in great effort to reach the goal. Although you fell a little short of the goal this week, keep pushing yourself this week. The goal will be increasing to 125 minutes. To help you achieve this goal, you could consider adding a positive social cue this week. Ask a friend, coworker or family member to join you on a walk, hike or bike ride. This is a great strategy for adding additional accountability and for making exercise fun and social.
Homework assignment completion	
Response option a: Assignment completed	It’s awesome to see that you have completed the homework this week! Hopefully, this homework will help you in the future to identify inactivity cues in your daily life and determine which ones you can replace with cues for activity. As you go about the week, keep thinking about ways that you can add more active cues to your home or work environment.
Response option b: Assignment not completed	A big part of this program is the homework that we assign each week. We think that it’s important to complete the assignment so that you can spend more time reflecting on what you learned in the lesson. You can still go back and complete the homework from last week. Take some time to do that now. Also, what got in the way of you completing the homework last week? If you simply forgot about it, try setting a reminder on your phone that prompts you at a certain time each week to complete your homework.
Atypical week	
Response option a: Injury	It looks like last week was a little atypical for you due to an injury. We hope that it isn’t anything serious and that you start to feel better soon! Please reach out to us if this injury persists.
Response option b: Illness	It looks like last week was a little atypical for you due to an illness. We hope that you are feeling better! Once you feel back to normal, make it a priority to get back to your exercise routine. Please let us know if your illness continues for over 2 weeks.
Response option c: Vacation	It looks like last week was a little atypical for you due to vacation. We hope that you had a great time! While vacation can sometimes be challenging because we get out of our routines, many times we can still find ways to be active while away. If you are still going to be on vacation this week, try to think of creative ways to exercise so that you can reach your goal. If you are home from vacation, make it a priority to get back in an exercise routine this week.

### Newsletter Condition

Participants randomized to the Newsletter condition, considered the usual care control condition, received newsletters twice per month for 3 months. Consistent with information available to individuals via the National Cancer Institute and traditional survivorship care plans, these newsletters focused on the general and cancer-specific health benefits of regular PA and reduced sedentary time and included recommended PA guidelines and general information for initiating aerobic PA, strength training, and flexibility programs. Other topics included were exercise safety, methods for gauging exercise intensity, an activity of the month (eg, Zumba, swimming, etc), and motivational, exercise-related stories from cancer survivors. Strategies for facilitating PA behavior change were not included in these newsletters.

### Assessment of PA

Moderate-to-vigorous physical activity (MVPA) was assessed at baseline, 3, and 6 months via both accelerometer and self-report. The Actigraph GT9X Link accelerometer [25-27] was worn on the waist for 7 consecutive days, during all waking hours (exclusive of bathing or water activities), at each assessment period. Participants were required to have ≥4 “valid” days (ie, ≥8 hours of wear time [28]) to be included in the analyses. A previously published cutpoint (≥1952 activity counts/min) was used to define MVPA [26] and weekly MVPA performed in bouts ≥1 minute (total MVPA) and ≥10 minutes (bouted MVPA) were computed using ActiLife software (version 6.13.5; ActiGraph, LLC).

Self-reported PA was assessed via a modified version of the Paffenbarger Physical Activity Questionnaire [29]. At each assessment period, participants were queried by research staff regarding their PA over the previous 7 days. Specifically, participants were asked to report the number of days and minutes per day spent (1) walking briskly for the purpose of exercise or transportation (eg, outside, indoors, or on a treadmill) for at least 10 consecutive minutes, and (2) engaging in sports and recreational activities, excluding occupational and household activities (eg, cleaning, laundry, and yard work). Minutes per week spent brisk walking and engaging in sports and recreational activities were summed to compute total minutes per week of MVPA.

### Questionnaire Measures

To address the secondary aim of comparing treatment groups on measures of physical and mental well-being, participants completed a series of questionnaires at each assessment period via REDCap (an online platform; Vanderbilt University). Health-related quality of life was assessed using the 36-Item Short Form Survey Instrument (SF-36) [30]. This previously validated and widely used questionnaire measures 8 domains of health status: physical functioning, physical role limitations, bodily pain, general health perceptions, vitality, social functioning, emotional role limitations, and mental health. Higher scores on each subscale indicate a more favorable quality of life. Fatigue was assessed using the Brief Fatigue Inventory, which has been previously validated in a cancer population [31]. This measure assesses the severity of fatigue and the impact of fatigue on daily functioning by querying individuals regarding fatigue over the past week, current fatigue, and usual and worst fatigue in the past 24 hours. An overall fatigue score was calculated, with higher scores indicating more severe fatigue. Psychological distress was assessed using the single-item Distress Thermometer developed by the National Comprehensive Cancer Network [32]. Individuals are asked to rate their distress on a scale of 0‐10, with scores ≥4 typically suggesting clinically significant distress. The Brief Symptom Inventory-18 [33] is widely used to assess psychological symptoms in cancer survivors and has 3 subscales (somatization, depression, and anxiety) and a global score (Global Severity Index, which reflects the overall level of psychological distress). Higher scores on all subscales indicate greater psychological distress. The Fear of Recurrence Inventory – Short Form [34] assessed the intensity of fear related to the possibility of cancer returning. A total score was computed, with higher scores reflecting a greater severity of fear of cancer recurrence.

### Statistical Analysis

The statistical analyses for this study were conducted using mixed-effects models to evaluate the impact of the program on various PA and psychological outcomes. The primary outcomes analyzed included self-reported MVPA, total MVPA, and bouted MVPA over time. Since this was a pilot study with a relatively small number of participants, we focus on effect sizes rather than *P* values.

For each outcome, mixed-effects models were used, incorporating random intercepts for participants to account for the repeated measures design of the study. These models included fixed effects for time points, treatment arms, and their interaction, as well as baseline values of each outcome to control for initial differences across groups. Additional covariates such as age, BMI, weight, sex, education, racial and ethnic minority status, maintenance therapy, days since treatment completion, and daily wear time (for accelerometer-based outcomes) were also included. All mixed-effects models were fitted using maximum likelihood estimation so that participants with incomplete data could be included with all available observations. This approach assumes that the data are missing at random, with missingness depending only on observed variables in the model (eg, baseline outcome, demographics, and clinical covariates). For accelerometer outcomes, analyses were restricted to assessments with ≥4 valid wear days, and daily wear time was included as a covariate. The primary interest was in the interaction between treatment arms and time points, which was used to evaluate whether changes in PA differed between the 2 groups (Energize! and Newsletter) at each time point (baseline, 3 months, and 6 months).

Post hoc pairwise comparisons of estimated marginal means were conducted to assess changes from baseline to 3 months and baseline to 6 months within each randomization group. Bonferroni corrections were applied to adjust for multiple comparisons. To further interpret the results, effect sizes were calculated using standardized mean differences (Cohen *d*), where 0.2 is considered a “small” effect, 0.5 is a “medium” effect, and 0.8 is a “large” effect.

### Ethical Considerations

Study procedures were approved by the Lifespan Institutional Review Board (Lifespan IRB 3, project # 1850077). Written or electronic informed consent was obtained by all participants prior to initiating any study procedures. All study data collected were deidentified and stored on a secure server. Participants were compensated US $25 in the form of cash or a gift card upon completion of assessment procedures at 3 and 6 months (for a total compensation of US $50). No compensation was provided for baseline assessments.

## Results

### Principal Results

**Figure 1.**
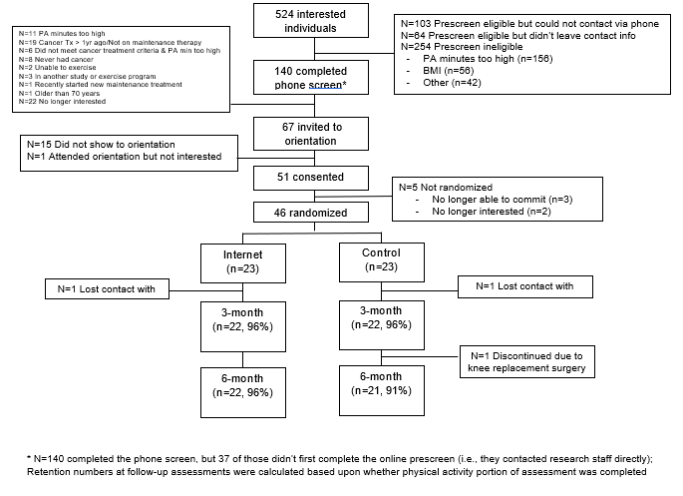
CONSORT (Consolidated Standards of Reporting Trials) diagram depicting patient flow through the trial.

Forty-six cancer survivors were randomized to the Energize! Program (n=23) or Newsletter condition (n=23; Figure 1). On average, participants were 55.2 (SD 8.3) years of age, had a BMI of 33.0 (SD 7.6) kg/m^2^, 91.3% self-reported as female (42/46), and 80.4% were non-Hispanic White (37/46). Among the sample, 69.6% were breast cancer survivors (32/46; vs “other” cancer type), 65.2% were on a maintenance therapy regimen (30/46), and on average participants were 684.7 (SD 861.5) days posttreatment. From a health literacy perspective, only 1 participant indicated “sometimes” having someone help them read instructions, pamphlets, or other written material from their doctor or pharmacy [35]. Participants also rated the importance of exercise in their lives as moderately high, mean 5.0/7.0 (SD 1.5), which was significantly higher than prior to their cancer diagnosis, mean 3.9 (SD 1.3; *P*<.001). There were no differences between treatment groups on any baseline demographic characteristics (Table 2).

**Table 2.** Comparison of baseline characteristics of study participants.^a^

Variables	Total (N=46)	Energize (n=23)	Newsletter (n=23)	*P* value
Age (years), mean (SD)	55.2 (8.3)	54.4 (7.9)	56.0 (8.8)	.52
BMI (kg/m^2^), mean (SD)	33.0 (7.6)	33.0 (9.4)	33.0 (5.4)	≥.99
Weight (kg), mean (SD)	88.5 (15.5)	87.8 (13.6)	89.2 (17.5)	.77
Biological sex, n (%)				.11
Female	42 (91.3)	19 (82.6)	23 (100.0)	
Male	4 (8.7)	4 (17.4)	0 (.0)	
Race and ethnicity, n (%)				.46
Non-Hispanic White	37 (80.4)	20 (87.0)	17 (73.9)	
Other	9 (19.6)	3 (13.0)	6 (26.1)	
Education, n (%)				.49
College degree or higher	35 (76.1)	19 (82.6)	16 (69.6)	
Less than college degree	11 (23.9)	4 (17.4)	7 (30.4)	
Cancer type, n (%)				.34
Breast cancer	32 (69.6)	14 (60.9)	18 (78.3)	
Other^b^	14 (30.4)	9 (39.1)	5 (21.7)	
Maintenance therapy, n (%)				≥.99
Yes	30 (65.2)	15 (65.2)	15 (65.2)	
No	16 (34.8)	8 (34.8)	8 (34.8)	
Duration since end of treatment (days), mean (SD)	684.7 (861.5)	665.0 (728.5)	703.6 (988.5)	.88
Ex importance prior to cancer	3.9 (1.3)	3.9 (1.3)	3.9 (1.4)	≥.99
Ex importance after cancer	5.0 (1.5)	4.7 (1.4)	5.2 (1.6)	.34

a Mean (SD) for continuous variables and n (%) for categorical variables.

b Other categories include: colorectal (n=2), esophageal (n=1), melanoma (n=2), liver (n=1), uterine (n=2), lung (n=2), more than one type (n=4).

### Feasibility and Acceptability Metrics

Study enrollment (n=46) was consistent with the targeted goal of approximately 50 participants. Feasibility metrics indicate that 48% of those who completed the screening process were deemed eligible (67/140), and of those who were deemed eligible, 69% were randomized (46/67). Further, retention at 3 and 6 months was 96% (44/46) and 94% (43/46), respectively. Acceptability was assessed via intervention engagement and program satisfaction. On average, Energize! participants watched 87.7% (SD 21.3%) video lessons (10.5/12), submitted an exercise plan on 82.6% (SD 25.8%) of weeks (9.9/12), completed 77.2% (SD 25.2%) of all homework assignments (9.3/12), logged their exercise minutes on 85.9% (SD 22.1%) of all weeks (10.3/12), and achieved their prescribed exercise goal on 56.5% of all weeks (6.8/12; weeks 1‐6: 70.3%, weeks 7‐12: 42.8%). When asked to rate the usefulness of each of the intervention elements (via Likert scales 1‐7), participants rated exercise planning (mean 5.0, SD 2.0) and exercise self-monitoring (mean 5.0, SD 2.0) as most useful, followed by video lessons (mean 4.7, SD 1.7), feedback messages (mean 4.3, SD 1.6), and homework assignments (mean 4.2, SD 1.8). Further, compared to the Newsletter condition, Energize! participants reported higher program satisfaction (mean 5.8, SD 1.6 vs mean 3.2, SD 1.6; *P*<.001), usefulness of the program (mean 5.6, SD 1.5 vs mean 4.2, SD 2.1; *P*<.001), and reported a higher likelihood of recommending the program to others (mean 5.9, SD 1.4 vs mean 4.8, SD 1.9; *P*=.001).

### Physical Activity Outcomes

**Figure 2.**
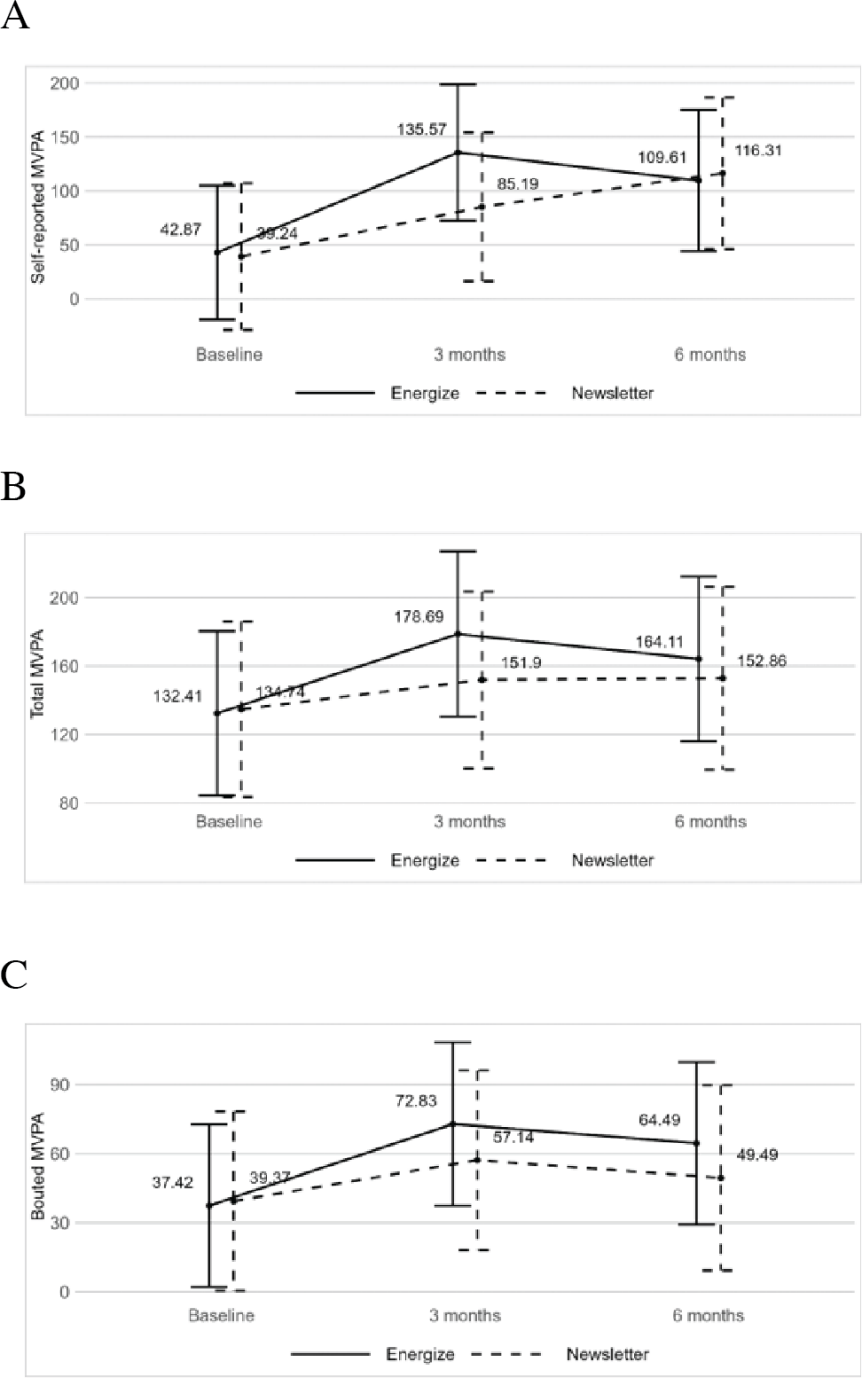
Change in (A) self-reported moderate-to-vigorous physical activity, (B) total moderate-to-vigorous physical activity, and (C) bouted moderate-to-vigorous physical activity over time by treatment arm. MVPA: moderate-to-vigorous physical activity.

On average, participants had 6.4 (SD 1.1) “valid” days of accelerometer wear time, and the device was worn for mean 13.3 (SD 1.7) hours/day across all time points. Changes in MVPA over time by treatment arm are shown in Figure 2. Mixed-effects models revealed that the interaction between randomization group and time was not significant for any of the MVPA variables from either baseline to 3 months (*P*>.14, *d*=0.36-0.51) or baseline to 6 months (*P*>.41, *d*=–0.07 to 0.34). However, effect sizes for the change in PA from baseline to 3 months for Energize! participants were in the medium-to-large range, with mean changes as follows: +92.7 minutes/week for self-reported MVPA (*P*=.01; *d*=0.94), +46.3 minutes/week for total MVPA (*P*=.06; *d*=0.74), and +35.4 minutes/week for bouted MVPA (*P*=.04; *d*=0.81). Among the newsletter conditions, no significant 3-month changes in MVPA were observed, and effect sizes were “small” for self-reported MVPA (+46.0 min/wk; *P*=.40; *d*=0.47), total MVPA (+17.2 min/wk; *P*≥.99; *d*=0.28), and bouted MVPA (+17.8 min/wk; *P*=.61; *d*=0.41). Changes from 3 to 6 months were not significant for either Energize! (*P*≥.99, *d*=0.19-0.26) or Newsletter (*P*>.95, *d*=0.02-0.32).

### Questionnaire Measures

Table 3 reports the mean scores for all questionnaire measures over the 6-month study period and compares treatment groups at 3 and 6 months. Comparisons between Energize! and Newsletter with medium-to-large effect sizes (eg, ≥0.5) are highlighted below. At 3 months, the vitality subscale of the SF-36 (*d*=0.63) and the somatization subscale of the Brief Symptom Inventory (*d*=0.76) were more favorable in Energize! compared to the Newsletter. At 6 months, outcomes favored Energize! over Newsletter for the depression (*d*=0.59) and anxiety subscales (0.51) of the Brief Symptom Inventory. However, Newsletter improved more than Energize! on the bodily pain (*d*=–0.85) and general health (*d*=–1.26) subscales of the SF-36 and on the Brief Fatigue Inventory (*d*=0.61).

**Table 3.** Questionnaire measure scores by treatment arm at baseline, 3, and 6 months.^a^

Variables	Baseline		3 months		6 months		*P* value (3 months)	Cohen *d* (3 months)	*P* value (6 months)	Cohen *d* (6 months)
	Energize (n=23)	Newsletter (n=23)	Energize (n=22)	Newsletter (n=22)	Energize (n=22)	Newsletter (n=19)				
Health-related quality of life, mean (SE)										
Physical functioning	78.74 (4.19)	77.64 (4.61)	80.36 (4.21)	81.63 (4.59)	78.88 (4.33)	81.51 (4.78)	.79	−0.10	.58	−0.20
Role physical	55.51 (9.55)	45.64 (10.69)	53.26 (9.6)	47.44 (10.7)	49.57 (9.95)	58.36 (11.37)	.61	0.18	.45	−0.28
Role emotional	65.92 (8.46)	58.15 (9.26)	81.94 (8.51)	77.17 (9.25)	72.05 (8.82)	69.26 (9.77)	.63	0.17	.79	0.10
Vitality	37.43 (4.37)	35.42 (4.74)	51.67 (4.4)	44.04 (4.71)	48.32 (4.51)	46.29 (4.86)	.10	0.63	.67	0.17
Mental health	69.66 (2.76)	68.39 (3.04)	76.89 (2.77)	74.75 (3.03)	77.51 (2.85)	74.5 (3.14)	.48	0.27	.33	0.37
Social functioning	69.67 (4.61)	63.4 (5.03)	78.92 (4.63)	73.76 (5.03)	79.98 (4.8)	78.43 (5.29)	.34	0.34	.78	0.10
Bodily pain	66.87 (4.67)	60.63 (5.37)	67.65 (4.68)	66.11 (5.4)	59.92 (4.82)	71.9 (5.6)	.78	0.11	.04	−0.85
General health	56.28 (2.76)	56.01 (3.12)	57.86 (2.77)	59.6 (3.07)	55.5 (2.86)	65.48 (3.17)	.57	−0.22	.002	−1.26
Brief Fatigue Inventory	3.84 (0.43)	4.34 (0.47)	3.46 (0.44)	4 (0.47)	3.87 (0.44)	3.06 (0.49)	.28	−.41	.12	.61
Psychological distress	2.24 (0.53)	2.31 (0.61)	2.32 (0.55)	2.24 (0.61)	2.03 (0.56)	1.54 (0.63)	.89	0.05	.46	0.28
Brief Symptom Inventory										
Somatization	3.46 (0.64)	3.75 (0.73)	3.42 (0.65)	4.71 (0.73)	3.93 (0.65)	3.44 (0.75)	.06	−0.76	.48	0.29
Depression	3.28 (0.77)	3.95 (0.9)	3.4 (0.78)	3.51 (0.89)	2.05 (0.79)	3.37 (0.92)	.91	−0.05	.14	−0.59
Anxiety	2.82 (0.62)	2.76 (0.69)	2.75 (0.64)	2.75 (0.68)	2.03 (0.65)	3.01 (0.71)	≥.99	0.00	.17	−0.51
Global	9.69 (1.5)	10.71 (1.71)	9.7 (1.52)	11.19 (1.7)	8.07 (1.54)	10.07 (1.76)	.38	−0.33	.25	−0.44
Fear of cancer recurrence	13.55 (1)	14.23 (1.1)	11.94 (1.01)	13.04 (1.1)	12.17 (1.03)	12 (1.14)	.32	−0.38	.88	0.06

a For all health-related quality of life subscales, higher scores are indicative of a more favorable outcome; however, higher scores for fatigue, psychological distress, brief symptom inventory, and cancer recurrence are indicative of a less favorable outcome.

## Discussion

### Principal Findings

Results of this preliminary investigation support the feasibility and short-term efficacy of a 3-month, fully automated, web-based intervention (Energize!) for improving MVPA among cancer survivors. Participant retention (>94%), weekly engagement with intervention elements (73%‐86%), and program satisfaction ratings (average of 5.8 out of 7) were high. Additionally, the Energize! program resulted in significant increases in both self-reported and accelerometer-derived MVPA from baseline to 3 months. Although there was no statistical difference between treatment groups, effect sizes for MVPA were in the small-to-medium range, favoring Energize! over the newsletter condition. At 6 months, there was little difference in MVPA between treatment groups.

### Comparison With Prior Work

Although changes in PA between treatment groups were not statistically significant, within the Energize! group, participants significantly increased their MVPA from baseline to 3 months by 93 minutes/week for self-reported PA and 35 and 46 minutes/week for accelerometer-derived bouted and total MVPA, respectively. This magnitude of change is in line with prior reports of PA promotion interventions among cancer survivors [36-39], and has been suggested to be clinically meaningful for overall health and well-being [40]. Furthermore, self-reported increases in PA, as small as 35 minutes/week, have been shown to reduce cancer-specific and all-cause mortality, underscoring the relevance of the results from this investigation [41]. Finally, it should be noted that the improvements in PA observed within the Energize! group were partially maintained at 6 months, and full recidivism to baseline levels did not occur, despite the absence of any additional intervention during 3 to 6 months. While this recidivism is disappointing, these findings are consistent with the broader PA literature, which demonstrates the challenge of maintaining PA following an unsupervised exercise program [42,43]. Future investigations may want to consider extending the length of the active intervention period, adding additional behavior change strategies that specifically target habit formation or maintenance, or examining state and trait level differences between those who successfully maintain PA postintervention versus those who do not.

While effect size estimates indicate that changes in PA were larger with Energize! compared to the newsletter condition, the mean change in MVPA in the newsletter group ranged from 17 to 46 minutes/week (depending on the measurement method). This was somewhat surprising, as newsletter participants did not receive behaviorally-based PA promotion content. Further, we asked participants at 3 months whether they signed up for any formal exercise programs, and only 1 participant indicated joining a weight loss program. There may be several explanations for the higher-than-expected PA in the Newsletter condition. First, it is possible that being asked to wear a PA monitor and answer questions related to PA could have prompted greater PA, as participants may have gleaned the importance of this behavior for study outcomes [44]. Alternatively, there are some data to suggest that cancer survivors are often unaware of, confused by, or not informed of PA recommendations during cancer aftercare [45,46]; thus, providing even the most basic information regarding the benefits of PA may have been sufficient to stimulate some level of behavior change. Finally, it is also possible that motivation for increasing PA is already very high among cancer survivors who make the decision to sign up for a PA program. This is corroborated by past research in which participants were randomized to a wait-list control condition, yet improvements in PA were still observed [41]. Although we cannot pinpoint the exact reason for the increase in PA levels in the Newsletter condition, these findings highlight the need for further research on PA behavior change during cancer survivorship.

Another interesting, yet not surprising, finding was the discordance between self-reported and accelerometer-derived PA. The results from this study are consistent with prior research, in that accelerometer-derived bouted MVPA was lower than self-reported MVPA [47,48]. There may be several explanations for this incongruency. First, although the monitors used in this study were research grade, these waist-worn activity monitors do not capture all forms of activity (ie, cycling, swimming, water aerobics, etc) [41,49]. Our self-report data suggest that 7%, 16%, and 19% of participants engaged in these types of activities at baseline, 3, and 6 months, respectively. In addition, cutpoints used for classifying accelerometer-derived MVPA were established via validation studies in healthy individuals, yet cancer survivors often have lower fitness levels and deconditioning due to treatment or fatigue. While it could be possible that activities that were indeed “moderate” based upon a participant’s fitness level fell below the accelerometer threshold for MVPA, our data do not support this hypothesis. We conducted exploratory analyses which found that there were no differences in light intensity PA or total PA (which combines light, moderate, and vigorous PA) between Energize! and Newsletter participants from pre- to postintervention (data not presented). Future research should continue to obtain both self-report and device-measured assessments of activity to help garner a clearer understanding of PA behavior in this population [41,49].

Another aim of this study was to examine the effect of the Energize! Exercise program on common cancer-related mental and physical well-being outcomes. At 3 months, medium effect sizes, favoring Energize! over Newsletter, were observed for vitality (ie, energy and fatigue) and somatization (ie, the experience and expression of psychological distress through physical symptoms). At 6 months, mixed findings were observed, with some outcomes favoring Energize! (depression and anxiety) and others favoring Newsletter (bodily pain, general health, and fatigue). One potential explanation for these findings is that the magnitude of change in PA in Energize! (relative to Newsletter) was not sufficient to elicit changes in the majority of the health outcomes assessed. Another possibility is that baseline differences between treatment groups may have made it difficult for intervention effects to be observed (eg, the majority of health-related quality of life scores at baseline were significantly higher in Energize! than in Newsletter). Nonetheless, these mixed findings are similar to meta-analyses and systematic reviews of digital PA promotion interventions on health-related outcomes in cancer survivors, which have also reported mixed results [23,24,39]. Future studies are needed to better understand factors contributing to these discrepant findings and to determine which behavior change techniques are most effective within web-based interventions for modifying mental and physical well-being outcomes.

### Limitations

This study has notable strengths, which include the use of both self-report and accelerometer-derived PA measurements, a no-contact intervention follow-up period to assess the lasting effect of the intervention, and the inclusion of individuals with varying types of cancer, which enhances generalizability. However, it is not without limitations. First, this was a preliminary investigation examining the effect of the Energize! program on MVPA and cancer-related mental and physical well-being; thus, it was not powered to detect differences between treatment groups. While effect size estimates are initially favorable regarding the Energize! program, a fully powered trial is warranted. Further, the majority of the participants in this study were female and non-Hispanic White. This is common to both the broader eHealth literature and cancer-specific literature [43,50], and limits the generalizability of findings. Finally, while inclusion criteria required participants to report engaging in less than 60 minutes of PA over the past 3 months on the initial eligibility screener, some participants were above this threshold at baseline. This suggests that participants may have been highly motivated and begun to increase PA prior to the start of the intervention, which could also have attenuated the intervention effect. Finally, participants were in different stages of survivorship when enrolling in this study; therefore, it’s possible that this could have affected rates of program engagement and changes in MVPA. Larger studies are needed to determine if individualized tailoring could improve outcomes.

### Conclusions

This study supports the feasibility, acceptability, and preliminary efficacy of the Energize! Exercise Program, a 3-month remotely delivered PA promotion intervention for cancer survivors. Results of the investigation indicate participants were satisfied with the program, routinely engaged with all study elements, and the majority were retained for the entire study duration. PA significantly increased from baseline to 3 months among Energize! participants, and these increases were partially maintained at 6 months. Future studies should investigate whether additional components can be added to the Energize! program to enhance PA or health-related outcomes, and whether specific behavior change techniques could be used to promote the long-term maintenance of PA changes.

## Supplementary material

10.2196/79610 Checklist 1 CONSORT-eHEALTH checklist (V 1.6.2).

## Abbreviations

MVPA: moderate-to-vigorous physical activity
PA: physical activity
SF-36: 36-Item Short Form Survey Instrument
