# Supplementary material for: Evaluation of a Translatable Web-Based Intervention for Increasing Physical Activity Among Cancer Survivors: Pilot Randomized Trial
Source: JMIR Cancer. 2025 Oct 2;11:e79610. doi: 10.2196/79610 (PMC12490769; doi:10.2196/79610)
Supplement: Checklist 1 [file cancer-v11-e79610-s001.pdf]

CONSORT-EHEALTH Checklist V1.6.2 Report	Manuscript Number	79610
**(based on CONSORT-EHEALTH V1.6), available at [http://tinyurl.com/consort-ehealth-v1-6].**		
**Date completed**		
7/3/2025 11:53:33		
**by**		
Jessica Unick		
Evaluation of a translatable web-based intervention for increasing physical activity among cancer survivors: A pilot randomized trial		
TITLE		
**1a-i) Identify the mode of delivery in the title**		
web-based intervention for increasing physical activity'		
**1a-ii) Non-web-based components or important co-interventions in title**		
**1a-iii) Primary condition or target group in the title**		
"among cancer survivors"		
ABSTRACT		
**1b-i) Key features/functionalities/components of the intervention and comparator in the METHODS section of the ABSTRACT**		
"The Energize! Program involved weekly behaviorally-based video lessons, homework assignments, exercise planning and reporting, and progressive PA goals (75 to 200 min/week). Automated, personalized feedback was provided. The newsletter group received bi-monthly PA education newsletters (total of 6)"		
**1b-ii) Level of human involvement in the METHODS section of the ABSTRACT**		
"automated Internet program", "automated personalized feedback"		
**1b-iii) Open vs. closed, web-based (self-assessment) vs. face-to-face assessments in the METHODS section of the ABSTRACT**		
**1b-iv) RESULTS section in abstract must contain use data**		
"46 adults", "Retention rates (>94%) and compliance to the Energize! program (73-86%) were excellent. Energize! increased self-reported (92.7 min/week), bouted (35.4 min/week), and total (46.3 min/wk) MVPA at 3 months (Cohen's d=0.74-0.94), and these changes were partially maintained at 6 months. Increases in MVPA were smaller among Newsletter participants (Cohen's d=0.28-0.47)."		
**1b-v) CONCLUSIONS/DISCUSSION in abstract for negative trials**		
INTRODUCTION		
**2a-i) Problem and the type of system/solution**		
"Physical activity (PA) is a modifiable lifestyle behavior which can help ameliorate these adverse health effects and is positively associated with numerous physiological and psychological health benefits. However, estimates suggest that only 17 to 58% of cancer survivors achieve the national physical activity guideline of ≥150 min/week of moderate-intensity PA [13-19]. Thus, translatable interventions for increasing PA among the growing number of cancer survivors are needed."		
**2a-ii) Scientific background, rationale: What is known about the (type of) system**		
"Although eHealth interventions are widely used to promote PA and other lifestyle behaviors in 'non-clinical' settings or within certain chronic disease populations (e.g., cardiovascular disease, diabetes), application of these technologies to promote PA among cancer survivors has lagged behind [21]. Considerable progress has been made in the past several years, however there remains a shortage of randomized controlled trials, with stringent investigative processes, evaluating the effects of behavioral eHealth interventions among cancer survivors [22-24]; additional research in clearly warranted. "		
**Does your paper address CONSORT subitem 2b?**		

"This randomized trial examines the feasibility and acceptability of a 12-week, fully-automated Internet program for increasing PA among cancer survivors and explores the effect of this program on PA relative to a newsletter control condition. Specifically, it uses rigorous methodology (e.g., randomized trial and PA assessment via accelerometers), incorporates essential behavior change techniques (e.g., goal setting, action planning, self-monitoring, problem solving, and affect regulation strategies), and examines whether intervention effects are sustained 3 months post-intervention, thereby addressing important deficits in the current body or research. A secondary aim of this trial was to compare treatment groups on measures of physical and mental well-being over the entire study period."		
METHODS		
**3a) CONSORT: Description of trial design (such as parallel, factorial) including allocation ratio**		
"Forty-six cancer survivors were enrolled in this study and randomized to receive the 12-week Energize! Exercise Program or a Newsletter control condition. Randomization occurred at baseline using sealed, opaque envelopes containing pre-generated allocation assignments, stratified by maintenance therapy status (on vs. off therapy) to ensure balance across conditions. Envelopes were prepared in advance, sequentially numbered, and opened in order of enrollment. "		
**3b) CONSORT: Important changes to methods after trial commencement (such as eligibility criteria), with reasons**		
No changes to methods were made		
**3b-i) Bug fixes, Downtimes, Content Changes**		
**4a) CONSORT: Eligibility criteria for participants**		
"To be eligible, individuals needed to have a confirmed cancer diagnosis and have either completed all cancer-directed treatment in the past 3-12 months or be on a maintenance treatment regimen for which they have been stable for at least 3 months. Other eligibility criteria included being 18-70 years of age, a body mass index of 18.5 to <45 kg/m2, daily Internet access, English speaking, and inactive, defined as engaging in <60 min/week of self-reported moderate-intensity PA over the past 3 months. Exclusion criteria included current, recent, or planned pregnancy over the next 6 months, recent (< 2 years) hospitalization for a psychiatric condition, or presence of medical condition for which PA is contraindicated. These broad inclusion criteria were selected to enhance generalizability of study findings, and not limit enrollment to a particular cancer type."		
**4a-i) Computer / Internet literacy**		
**4a-ii) Open vs. closed, web-based vs. face-to-face assessments:**		
"These sessions were conducted in person or via videoconference (e.g., Zoom). "		
**4a-iii) Information giving during recruitment**		
"Participants were recruited via advertisements at local oncology clinics in the greater Providence RI area, and via national social media advertisements (e.g., Facebook). Interested individuals could scan a QR code on physical flyers or click on a link within a social media advertisement to be directed to an online screener. This screener was designed to assess initial eligibility by confirming These sessions were conducted in person or via videoconference (e.g., Zoom). that age, body mass index, ability to exercise, and physical activity criteria were met. Individuals deemed initially eligible based upon the online screener were then contacted by a member of the research team, and a more thorough eligibility screener was conducted via telephone. Those who continued to be eligible were invited to a one-on-one orientation session to learn more about the study, ask questions, and obtain informed consent. "		
**4b) CONSORT: Settings and locations where the data were collected**		
"Lifespan institutional review board"		
**4b-i) Report if outcomes were (self-)assessed through online questionnaires**		
"Assessments, which consisted of online questionnaires"		
**4b-ii) Report how institutional affiliations are displayed**		
**5) CONSORT: Describe the interventions for each group with sufficient details to allow replication, including how and when they were actually administered**		
**5-i) Mention names, credential, affiliations of the developers, sponsors, and owners**		
**5-ii) Describe the history/development process**		
**5-iii) Revisions and updating**		

**5-iv) Quality assurance methods**		
**5-v) Ensure replicability by publishing the source code, and/or providing screenshots/screen-capture video, and/or providing flowcharts of the algorithms used**		
**5-vi) Digital preservation**		
**5-vii) Access**		
This was not a commercially available application. Participants were provided access to the web-based platform as part of the study.		
**5-viii) Mode of delivery, features/functionalities/components of the intervention and comparator, and the theoretical framework**		
"Participants randomized to the intervention group received the 12-week Energize! Exercise Program (EEP). This fully automated, behaviorally-based, Internet program was designed to increase moderate-intensity PA to a level consistent with national recommendations. The entire program was delivered via a study website, which could be accessed via any web browser on a computer, tablet, or smartphone. No human intervention contact was provided. Each week, participants were given a prescribed exercise goal, and asked to submit an exercise plan, watch a multi-media lesson, complete a brief homework assignment, and report their exercise on the study website. Computer-generated, personalized feedback messages were also provided weekly. Each of the Energize! Program components are described in greater detail below.<br>Exercise goals. Participants were given a weekly, moderate-intensity PA goal that started at 75 min/week and increased by 25 minutes/week every other week, until reaching 200 minutes by week 11. Given that this program was designed to increase 'purposeful' moderate-intensity aerobic exercise (e.g., brisk walking, cycling), participants were instructed not to count activities such as hatha yoga, strength training, household chores (e.g., gardening or vacuuming), or occupational activities (e.g., restaurant server, postal worker), towards their weekly aerobic PA minute goal. To promote a regular habit of PA, participants were encouraged to exercise 5 days/week.<br>Exercise planning. Beginning at week 2, participants were encouraged to plan their exercise prior to the start of each week and submit that detailed exercise plan via the study website. Specifically, individuals were asked to consider their schedule for the upcoming week and record when they planned to exercise (i.e., day of week and time of day), as well as the type and duration of exercise they planned to do.<br>Multi-media lessons. Each week, participants were instructed to watch a 10-15 minute video lesson. These lessons were designed to teach behavioral principles for modifying PA behavior. During week 1, a general overview of the program was provided along with an exercise prescription. Other video lessons topics included exercise planning, managing negative thoughts, stimulus control, overcoming exercise barriers, affect and exercise enjoyment, a mid-point check-in, managing exercise slips, exercise motivation, turning sitting time into active time, future-oriented mindsets, and thinking like an exerciser.<br>Homework assignments. Participants were asked to complete weekly homework assignments online to help apply the content from the video lessons to their personal lives. Each assignment was designed to correspond to the lesson of the week and take no longer than 10 minutes to complete. Example assignments included: the creation of an exercise routine to promote habit formation, journaling about the value of exercise and positive feelings associated with exercise, problem solving around exercise barriers, and personal self-reflection based upon one's progress in the program. No feedback was provided to the participant related to their homework assignment responses.<br>Self-monitoring of exercise. Similar to the exercise planning component of this program, participants were also asked to report all exercise performed on the study website daily. This included the time of day that the exercise was performed, the type of exercise, and number of exercise minutes.<br>Automated feedback messages. Computer-generated personalized feedback was provided weekly based upon the data that was input from the previous week. Feedback messages took into consideration three factors: 1) number of exercise minutes performed, 2) whether the homework assignment was completed or not, and 3) whether any injury, illness, or vacation prevented the participant from achieving their weekly exercise goal. Feedback messages were designed to be encouraging and motivational, praising individuals for meeting goals, and providing support, encouragement, and specific behavioral recommendations for when goals were not met"		
**5-ix) Describe use parameters**		
**5-x) Clarify the level of human involvement**		
**5-xi) Report any prompts/reminders used**		
"Computer-generated personalized feedback was provided weekly based upon the data that was input from the previous week. Feedback messages took into consideration three factors: 1) number of exercise minutes performed, 2) whether the homework assignment was completed or not, and 3) whether any injury, illness, or vacation prevented the participant from achieving their weekly exercise goal. "		
**5-xii) Describe any co-interventions (incl. training/support)**		

This was a stand alone intervention		
**6a) CONSORT: Completely defined pre-specified primary and secondary outcome measures, including how and when they were assessed**		
"Moderate-to-vigorous intensity physical activity (MVPA) was assessed at baseline, 3, and 6 months via both accelerometer and self-report. The Actigraph GT9X Link accelerometer (Pensacola, FL)[25-27] was worn on the waist for 7 consecutive days, during all waking hours (exclusive of bathing or water activities), at each assessment period. Participants were required to have ≥4 'valid' days (i.e., ≥8 hours of wear time) to be included in the analyses. A previously published cutpoint (≥1952 activity counts/min) was used to define MVPA [26] and weekly MVPA performed in bouts ≥1 min (total MVPA) and ≥10 minutes (bouted MVPA) were computed using ActiLife software (version 6.13.5)."		
**6a-i) Online questionnaires: describe if they were validated for online use and apply CHERRIES items to describe how the questionnaires were designed/deployed**		
**6a-ii) Describe whether and how "use" (including intensity of use/dosage) was defined/measured/monitored**		
**6a-iii) Describe whether, how, and when qualitative feedback from participants was obtained**		
**6b) CONSORT: Any changes to trial outcomes after the trial commenced, with reasons**		
"Lifespan institutional review board"		
**7a) CONSORT: How sample size was determined**		
**7a-i) Describe whether and how expected attrition was taken into account when calculating the sample size**		
**7b) CONSORT: When applicable, explanation of any interim analyses and stopping guidelines**		
"Moderate-to-vigorous intensity physical activity (MVPA) was assessed at baseline, 3, and 6 months via both accelerometer and self-report. The Actigraph GT9X Link accelerometer (Pensacola, FL)[25-27] was worn on the waist for 7 consecutive days, during all waking hours (exclusive of bathing or water activities), at each assessment period. Participants were required to have ≥4 'valid' days (i.e., ≥8 hours of wear time) to be included in the analyses. A previously published cutpoint (≥1952 activity counts/min) was used to define MVPA [26] and weekly MVPA performed in bouts ≥1 min (total MVPA) and ≥10 minutes (bouted MVPA) were computed using ActiLife software (version 6.13.5)."		
**8a) CONSORT: Method used to generate the random allocation sequence**		
"Randomization occurred at baseline using sealed, opaque envelopes containing pre-generated allocation assignments, stratified by maintenance therapy status (on vs. off therapy) to ensure balance across conditions. Envelopes were prepared in advance, sequentially numbered, and opened in order of enrollment. "		
**8b) CONSORT: Type of randomisation; details of any restriction (such as blocking and block size)**		
"stratified by maintenance therapy status (on vs. off therapy) to ensure balance across conditions."		
**9) CONSORT: Mechanism used to implement the random allocation sequence (such as sequentially numbered containers), describing any steps taken to conceal the sequence until interventions were assigned**		
"opaque envelopes containing pre-generated allocation assignments"		
**10) CONSORT: Who generated the random allocation sequence, who enrolled participants, and who assigned participants to interventions**		
The PI generated the envelopes and research staff opened the envelopes in front of participants.		
**11a) CONSORT: Blinding - If done, who was blinded after assignment to interventions (for example, participants, care providers, those assessing outcomes) and how**		
**11a-i) Specify who was blinded, and who wasn't**		
Neither outcome assessors or participants were blinded.		
**11a-ii) Discuss e.g., whether participants knew which intervention was the "intervention of interest" and which one was the "comparator"**		
**11b) CONSORT: If relevant, description of the similarity of interventions**		
This question is not relevant to the this study		
**12a) CONSORT: Statistical methods used to compare groups for primary and secondary outcomes**		

"The statistical analyses for this study were conducted using mixed-effects models to evaluate the impact of the program on various physical activity and psychological outcomes. The primary outcomes analyzed included self-reported moderate-to-vigorous physical activity (MVPA), total MVPA, and bouted MVPA over time. Since this was a pilot study with a relatively small number of participants, we focus on effect sizes rather than p-values.<br>For each outcome, mixed-effects models were employed, incorporating random intercepts for participants to account for the repeated measures design of the study. These models included fixed effects for time points, treatment arms, and their interaction, as well as baseline values of each outcome to control for initial differences across groups. Additional covariates such as age, BMI, weight, sex, education, racial/ethnic minority status, maintenance therapy, days since treatment completion, and daily wear time (for accelerometer-based outcomes) were also included. The primary interest was in the interaction between treatment arms and time points, which was used to evaluate whether changes in physical activity differed between the two groups (Energize! and Newsletter) at each time point (baseline, 3 months, and 6 months).<br>Post hoc pairwise comparisons of estimated marginal means (EMMs) were conducted to assess changes from baseline to 3 months and baseline to 6 months within each randomization group. Bonferroni corrections were applied to adjust for multiple comparisons. To further interpret the results, effect sizes were calculated using standardized mean differences (Cohen's d), where 0.2 is considered a 'small' effect, 0.5 is a 'medium' effect, and 0.8 is a 'large' effect."		
**12a-i) Imputation techniques to deal with attrition / missing values**		
"The statistical analyses for this study were conducted using mixed-effects models to evaluate the impact of the program on various physical activity and psychological outcomes."		
**12b) CONSORT: Methods for additional analyses, such as subgroup analyses and adjusted analyses**		
We did not further subgroup analyses than what is mentioned above.		
RESULTS		
**13a) CONSORT: For each group, the numbers of participants who were randomly assigned, received intended treatment, and were analysed for the primary outcome**		
These are all included in the Consort figure included in the manuscript		
**13b) CONSORT: For each group, losses and exclusions after randomisation, together with reasons**		
See Consort figure included in paper		
**13b-i) Attrition diagram**		
**14a) CONSORT: Dates defining the periods of recruitment and follow-up**		
These have not been included within the paper which has already been submitted, but it was a 6-month study and all participants were recruited within a year.		
**14a-i) Indicate if critical "secular events" fell into the study period**		
**14b) CONSORT: Why the trial ended or was stopped (early)**		
N/A - the trial did not end early		
**15) CONSORT: A table showing baseline demographic and clinical characteristics for each group**		
This can be found in Table 1		
**15-i) Report demographics associated with digital divide issues**		
many of these factors are included in table 1.		
**16a) CONSORT: For each group, number of participants (denominator) included in each analysis and whether the analysis was by original assigned groups**		
**16-i) Report multiple "denominators" and provide definitions**		
The sample size (n) is included n all tables and within the text of the manuscript		
**16-ii) Primary analysis should be intent-to-treat**		
**17a) CONSORT: For each primary and secondary outcome, results for each group, and the estimated effect size and its precision (such as 95% confidence interval)**		

"Changes in physical activity over time by treatment arm are shown in Figure 2. Mixed effects models revealed that the interaction between randomization group and time was not significant for any of the MVPA variables from either baseline to 3 months (p's > 0.14, d's=0.36 to 0.51) or baseline to 6 months (p's>0.41, d's=-0.07 to 0.34). However, effect sizes for the change in PA from baseline to 3 months for Energize! participants were in the medium-to-large range, with mean changes as follows: +92.7 minutes/week for self-reported MVPA (p = 0.01; d=0.94), +46.3 minutes/week for total MVPA (p = 0.06; d=0.74), and +35.4 minutes/week for bouted MVPA (p = 0.04; d=0.81). Among the newsletter condition, no significant 3-month changes in MVPA were observed, and effect sizes were 'small' for self-reported MVPA (+46.0 min/week; p=0.40; d=0.47), total MVPA (+17.2 min/week; p=1.00; d=0.28), and bouted MVPA (+17.8 min/week; p=0.61; d=0.41). Changes from 3 to 6 months were not significant for either Energize! (p's=1.00, d's=0.19 to 0.26) or Newsletter (p's>0.95, d's=0.02 to 0.32). "		
**17a-i) Presentation of process outcomes such as metrics of use and intensity of use**		
**17b) CONSORT: For binary outcomes, presentation of both absolute and relative effect sizes is recommended**		
We did not have any binary outcomes.		
**18) CONSORT: Results of any other analyses performed, including subgroup analyses and adjusted analyses, distinguishing pre-specified from exploratory**		
Analyses are described as either primary or secondary.		
**18-i) Subgroup analysis of comparing only users**		
**19) CONSORT: All important harms or unintended effects in each group**		
There were no serious adverse events resulting from this trial		
**19-i) Include privacy breaches, technical problems**		
**19-ii) Include qualitative feedback from participants or observations from staff/researchers**		
DISCUSSION		
**20) CONSORT: Trial limitations, addressing sources of potential bias, imprecision, multiplicity of analyses**		
**20-i) Typical limitations in ehealth trials**		
"This study has notable strengths, which include the use of both self-report and accelerometer-derived PA measurements, a no-contact intervention follow-up period to assess the lasting effect of the intervention, and the inclusion of individuals with varying types of cancer, which enhances generalizability. However, it is not without limitations. First, this was a preliminary investigation examining the effect of the Energize! program on MVPA and cancer-related mental and physical well-being; thus, it was not powered to detect differences between treatment groups. While effect size estimates are initially favorable regarding the Energize! program, a fully-powered trial is warranted. Further, the majority of the participants in this study were female and non-Hispanic White. This is common to both the broader eHealth literature and cancer specific literature5,9, and limits generalizability of findings. Finally, while inclusion criteria required participants to report engaging in less than 60 minutes of PA over the past 3 months on the initial eligibility screener, some participants were above this threshold at baseline. This suggests that participants may have been highly motivated and begun to increase PA prior to the start of the intervention, which could also have attenuated the intervention effect. "		
**21) CONSORT: Generalisability (external validity, applicability) of the trial findings**		
**21-i) Generalizability to other populations**		
**21-ii) Discuss if there were elements in the RCT that would be different in a routine application setting**		
**22) CONSORT: Interpretation consistent with results, balancing benefits and harms, and considering other relevant evidence**		
**22-i) Restate study questions and summarize the answers suggested by the data, starting with primary outcomes and process outcomes (use)**		
"Results of this preliminary investigation support the feasibility and short-term efficacy of a 3-month, fully automated, online intervention (Energize!) for improving MVPA among cancer survivors. Participant retention (>93%), weekly engagement with intervention elements (73-86%), and program satisfaction ratings (average of 5.8 out of 7) were high. Additionally, the Energize! program resulted in significant increases in both self-reported and accelerometer-derived MVPA from baseline to 3 months. Although there was no statistical difference between treatment groups, effect sizes for MVPA were in the small-to-medium range, favoring Energize! over the newsletter condition. At 6 months, there was little difference in MVPA between treatment groups."		
**22-ii) Highlight unanswered new questions, suggest future research**		

Other information		
**23) CONSORT: Registration number and name of trial registry**		
Clinicaltrials.gov: NCT1850077		
**24) CONSORT: Where the full trial protocol can be accessed, if available**		
The full trial protocol can be assessed on clinicaltrials.gov		
**25) CONSORT: Sources of funding and other support (such as supply of drugs), role of funders**		
This study was funded by the Legorreta Cancer Center at Brown University.		
**X26-i) Comment on ethics committee approval**		
**x26-ii) Outline informed consent procedures**		
**X26-iii) Safety and security procedures**		
**X27-i) State the relation of the study team towards the system being evaluated**		
